# Spatiotemporal molecular medicine: A new era of clinical and translational medicine

**DOI:** 10.1002/ctm2.294

**Published:** 2021-01-17

**Authors:** Xiangdong Wang, Jia Fan

**Affiliations:** ^1^ Department of Pulmonary and Critical Care Medicine Zhongshan Hospital Institute for Clinical Science Shanghai Institute of Clinical Bioinformatics Shanghai Engineering Research for AI Technology for Cardiopulmonary Diseases Jinshan Hospital Centre for Tumor Diagnosis and Therapy Shanghai Medical College Fudan University Shanghai China; ^2^ Department of Liver Surgery and Transplantation Liver Cancer Institute Zhongshan Hospital and Key Laboratory of Carcinogenesis and Cancer Invasion (Ministry of Education) Key Laboratory of Medical Epigenetics and Metabolism Institutes of Biomedical Sciences State Key Laboratory of Genetic Engineering Fudan University Shanghai China

Spatiotemporal molecular medicine is a new discipline of medicine to precisely understand the pathogenesis, history, and epidemiology, and aims to achieve prevention, diagnosis, and therapy at molecular levels for human diseases in multidimensional aspects related to a spatial and temporal context. The human body is a three‐dimensional object with the space of length, width, and height, while the spatiotemporal concept demonstrates a four‐dimensional continuum with the concept of time. Clinical spatialization of a disease should be considered at the levels of genetics (e.g., family tree, pedigree, germline heterogeneity), populations (e.g., regional distribution, epidemiological map), and individuals (e.g., inter‐ and intraorgan location and variation). Clinical temporalization covers the historical process and progression of disease occurrence and development, duration‐based alteration of clinical phenomes, and dynamics of patient responses to therapy. Clinical trans‐omics was proposed as a new approach to understand the disease from the cross‐points among different layers of networks generated from the integration of clinical phenomics with molecular multi‐omics.^1^ Clinical trans‐omics provide three‐dimensional information on clinical phenome(s) corresponding to molecular elements or the reverse, the understanding of molecule‐based dimension in correlation with phenomes, and the potential of disease phenome‐specific diagnostic biomarkers and therapeutic targets. In comparison, spatiotemporal molecular medicine presents a four‐dimensional and dynamical picture of the disease by integrating clinical spatialization, temporalization, phenome, and molecular multi‐omics for disease diagnosis, therapy, and prognosis. We propose that spatiotemporal molecular medicine will be an independent and merging discipline to meet the rapid development of medicine. The present editorial especially highlights spatiotemporal molecular medicine as a new vision and platform to understand the importance of spatial and temporal transcriptomics/proteomics, positioning of cell–cell interaction and communication, organogenesis, and organ development for diagnosis and therapy. We also address the difference between spatiotemporal medicine and spatiotemporal molecular medicine, potential availability of spatiotemporal molecular omics measurements and analyses, and need of artificial intelligence and computerized models.

Spatiotemporal molecular omics are a major part of spatiotemporal molecular medicine. For example, transcriptomic profiles of tissues, isolated single category of cells, or single cells demonstrate tissue‐ or cell‐specific transcriptional function as one‐dimensional information of mRNA expression. Ståhl et al. labeled arrayed reverse transcription primers with unique barcodes, in situ hybridized positioning mRNA on tissue sections with those primers, as well as visually and quantitively imaged and analyzed transcriptomic profiles on tissue surface, coined as spatial transcriptomics, in order to define the exact position of transcriptional alterations with the tissue and cells.^2^ This was a revolutionary development to clearly define the exact locations of cells with up‐/downregulated RNA expression and provide two‐dimensional information. Furthermore, analyses of single cell and spatial transcriptomics were developed by integration of isolated single‐cell RNA sequencing and spatial transcriptomics as a new approach to reconstruct tissue visualization and neighbor structure and define cell–cell interactions in tissues, spatial relations between cell locations and transcriptional phenomes, and evolutions of lineages and populations. Single cell and spatial transcriptomics were proposed to have special values to deeply understand mechanisms of cell function and pathogenesis of the disease and precisely identify spatial responses of target cells to therapy.^3^ In addition, spatiotemporal proteomics provide an in‐depth cell‐type‐ and region‐specific proteomic catalog of the tissue section using the peptide identifications from the soluble or insoluble proteomes by single‐run liquid chromatography–tandem mass spectrometry. Spatiotemporal proteomics demonstrated that three selected clusters showed distinct protein expression across four regions with functional brain region specificity, responsible for the progression of Huntington's disease.^4^

Spatiotemporal molecular medicine has a high scientific impact on investigation of cell–cell communication, microenvironmental function, and interactomes. The interaction networks of genes, proteins, and cells within the microenvironment play a decisive role in the production of chemoattractants for inflammatory cell recruitment and growth factor production for cancer cell extensions. For example, the liver microenvironment was considered as one of the easy locations for extrahepatic tumor metastasis^5^, ^6^ due to rich contents of acellular components for metastatic niche formation, special phenomic profiles of cellular components and cell‐derived exosomes for the communication between resident cells, immigrated cancer cells and cancer stem cells, and dynamic processes of epithelial‐to‐mesenchymal or mesenchymal‐to‐epithelial transitions. With the rapid development of spatiotemporal measurements and analyses, we can define and monitor spatiotemporal gradient formation of acellular components, dynamic intra‐/intercellular communications and regulations of cell signals, and dynamics of cancer stem cell heterogeneity and intratumoral immune cell landscape. In addition to spatial transcriptomics and proteomics, spatiotemporal microenvironment can be investigated by spatiotemporal target‐paneled multi‐omics, three‐dimensional in vitro model of cell‐engineered microenvironment, and spatiotemporal nanoparticle transitions.

Spatiotemporal molecular medicine has the special power to explore molecular mechanisms and modifications of embryonic development, tissue morphogenesis, and organogenesis. Genomic dimensions, architectures, and organization play critical roles in regulations of gene transcription, spatiotemporal development, genome assembly, and target‐based therapy.^7^ The developmental dynamics and regulations of multiple foci/cell clusters and regeneration from the same or different clones can be geographically and temporally dependent upon genomic characterization. The heterogeneity of genomic organizations exists in normal development and tumor growth/recurrence and is responsible for cancer cell responses to therapy. Lee et al. measured genomic and expression signatures of bulk cells, single cells, and selected regional cells isolated from tumor tissues and found the therapeutic response of glioma cells depended upon genetic similarity, mutations, and heterogeneities.^8^ Spatiotemporal molecular medicine could provide multidimensional insights into organogenesis during which gene regulations, protein interactions, and cell–cell communications in special positions are dynamically monitored for understanding mechanisms and prediction of diseases. It is possible to dynamically uncover the correlation of chromatin rearrangement with transcriptional networks, regulation of multiple factors at various levels to control cell fate and differentiation as well as rearrangement, and formation of organ‐specific cell phenomes and function in specific cells. It allows for precise identification of the occurrence of dysfunction in signaling molecules, differentiation processes, cell types, and locations within the tissue architecture. From spatiotemporal morphogenesis, precision medicine will be further developed and improved for clinical diagnosis and therapy.

Spatiotemporal medicine exists in clinic diagnosis, therapy, and prognosis for decades. The process of clinically examining responses of different organs to pathogens, diseases, and therapies at various stages and durations per se is a spatiotemporal course. Spatiotemporal images of organ can be constructed using ultrasounds, computed tomography (CT), nuclear magnetic resonance/CT, positron‐emission tomography/CT, or pathological sections. Of those, four‐dimensional ultrasound using spatiotemporal image correlation with software allows for detection of organ anatomy and function during aging and has special impact in disease diagnosis. Fluorescence endomicroscopy with automated image analysis can produce large quantities of data on the spatiotemporal behavior of target gene expression in cells of human digestive and respiratory tissues to analyze the geometry and fluorescence image and monitor temporal changes in gene expression. The temporal notion of causality between clinical phenomes observed over a period of time (e.g., symptoms, signs, images, biochemical measures, and responses to therapy) becomes an important part of spatiotemporal medicine, as suggested by considering shorter and longer time‐series.^9^ Spatiotemporally fractionated treatment plans of photon radiotherapy for liver cancer were suggested as an efficient approach to save healthy tissue from the therapeutic target area.^10^ Spatiotemporal epidemiology has been also applied for monitoring the frequency of the diseases, for example, glanders, mycorrhizal fungi, Ebola virus spillover, as well as environmental and socioeconomic factors.

Spatiotemporal molecular medicine contains an additional molecular level on top of spatiotemporal medicine and becomes appliable in clinical practice and investigation with the development of molecular barcode labeling technologies. Advanced methods to detect the spatial positioning of mRNA and protein expression in specific cells in tissue sections provide a new opportunity for defining the exact locations of disease‐, disfunction‐, signal‐, and regulation‐specific alterations in pathological conditions. This makes it possible to identify DNA methylation and transcriptomic information with protein expression in the immunophenotyping of cells on pathological images of ultrasounds and CT in the future, although many challenges remain to be overcome. The integration of spatiotemporal medicine with molecular spatial‐omics maps can provide new strategies for early diagnosis and individualized therapies at molecular levels. The methodologies to merge image profiles and clinical phenomes with molecular profiles include in situ hybridization/fluorescence staining with oligonucleotide probes, digital barcodes tag with oligonucleotides, DNA microscopy with chemical DNA reactions fluorescent barcoding with sequencing, Drop‐seq technology integrating single‐cell RNA sequencing and imaging, padlock amplification of gene sequencing, cryogenic tissue sections with RNA sequencing and spatial data, cryogenic tissues from laser capture microdissection and RNA sequencing, cDNA synthesis in situ with spatial barcoding and RNA sequencing, mRNA barcoded in situ with spatial sequencing by oligonucleotide ligation and detection, virtual reconstruction of the tissue from single‐cell RNA sequencing, and new transcriptome alkylation‐dependent single‐cell RNA sequencing to identify temporal and spatial features of single‐cell data.^11^, ^12^, ^13^ The methods of spatial proteome represented by localizations and dynamics of proteins at subcellular level are potentials for global comparative applications to figure out cell variations, dynamic protein translocations, interaction networks, and compartments of protein location.^14^

Spatiotemporal molecular medicine is a part of molecular medicine characterized by gene‐based diagnosis and therapy. With the improvement of on‐/off‐target site specificity and efficiency and improved understanding of structures, mechanisms, clinical applications, and off‐target activities of genome editing systems, genome editing will become one of the clinical precision medicine strategies and multidisciplinary therapy strategies by integrating gene sequencing, clinical trans‐omics, and single‐cell biomedicine.^15^ As parts of spatiotemporal molecular medicine, spatial transcriptomics, proteomics, metabolomics, and bioinformatics can be the important tools to evaluate whether the body is suitable for targeted gene editing by programmable endonucleases prior to the treatment and monitor how the systems’ responses occur and how spatiotemporal controls might regulate functional switches or be regulated by gene editing associated alterations after the gene therapy.^16^, ^17^

Spatiotemporal molecular medicine requires the obvious contributions from rapid development of artificial intelligence, automatic robots, and computational and mathematical models. For example, the artificial intelligent cell was proposed as a system with computerized databases, digitalized informatics of biological elements, and programmed function and signals at a single‐cell level.^18^ Although the system is still under development, spatiotemporal artificial intelligent cells with deep learning and auto‐programming capacities, especially integrative capacity of clinical phenomic profiles, will make clinical application of spatiotemporal molecular medicine much easier. One of spatiotemporal molecular medicine aims is to translate spatiotemporal distributions of gene and protein profiles, regulations, and intercellular interaction and communication into clinical phenotypes, patient response to therapies, and strategies of precision medicine. The integration of single‐molecule spectroscopy, multi‐omic profiles, clinical phenomes, and numerical modeling is an approach to understand fluctuations of cell/organ responses and gene regulatory process from multiple single‐cell datasets. Many computerized models have been developed, including air pollution spatiotemporal model for monitoring public health, spatiotemporal chromatin fluidity model for detecting heterogeneity dynamics, population shape decoding model for neuron spatio‐temporal features, and network‐based bioinformatics model for spatiotemporal RNA sequencing data analyses. Spatiotemporal computerization of human organ anatomy and function for medical education and clinical operation is still in the process of maturing. Spatial navigation and reinforcement learning of the brain are modeled using descriptive, mechanistic, and normative approaches assisted by artificial intelligence, which enables spatial positioning of pathological focus, correspondence between neuro‐structure and behavior, and definition of molecular networks in neurons.^19^ As a new discipline, spatiotemporal molecular medicine faces many challenges, for example, how to clearly define and understand concepts, categorize contents, and standardize protocols of measurements and analyses.

In conclusion, we propose the concept of spatiotemporal molecular medicine as a new discipline of medicine and believe it will become more important and applicable with advanced development of spatial and temporal transcriptomics, proteomics, and metabolomics. Clinical spatialization and temporalization of phenomes are integrated with the information from spatiotemporal molecular omics to form a four‐dimensional understanding, diagnosis, and therapy for patients. Spatiotemporal measurements and analyses are decisive factors that may limit clinical applications of spatiotemporal molecular medicine. Although many challenges need to be overcome, spatiotemporal molecular medicine will improve our knowledge of diseases, provide multidimensional and targeted panels for more precise and early diagnosis and therapy, and be one of the future topics in clinical and translational medicine.
